# Hospital versus individual surgeon’s performance in laparoscopic hysterectomy

**DOI:** 10.1007/s00404-016-4199-2

**Published:** 2016-09-15

**Authors:** Sara R. C. Driessen, Markus Wallwiener, Florin-Andrei Taran, Sarah L. Cohen, Bernhard Kraemer, Christian W. Wallwiener, Erik W. van Zwet, Sara Y. Brucker, Frank Willem Jansen

**Affiliations:** 1 Department of Gynecology, Leiden University Medical Center, PO Box 9600, 2300 RC Leiden, The Netherlands; 2 Department of Obstetrics and Gynecology, University of Heidelberg, INF 440, 69115 Heidelberg, Germany; 3 Department of Obstetrics and Gynecology, University of Tuebingen, Calwerstr. 7, 72076 Tuebingen, Germany; 4 Division of Minimally Invasive Gynecologic Surgery, Brigham and Women’s Hospital, 75 Francis St, Boston, MA 02115 USA; 5 Department of Medical Statistics, Leiden University Medical Centre, PO Box 9600, 2300 RC Leiden, The Netherlands; 6 Department BioMechanical Engineering, Delft University of Technology, PO Box 5, 2600 AA Delft, The Netherlands

**Keywords:** Laparoscopic hysterectomy, Case-mix, Experience, Outcome, Volume, Hospital outcome

## Abstract

**Purpose:**

To compare hospital versus individual surgeon’s perioperative outcomes for laparoscopic hysterectomy (LH), and to assess the relationship between surgeon experience and perioperative outcomes.

**Methods:**

A retrospective analysis of all prospective collected LHs performed from 2003 to 2010 at one medical center was performed. Perioperative outcomes (operative time, blood loss, complication rate) were assessed on both a hospital level and surgeon level using Cumulative Observed minus Expected performance graphs.

**Results:**

A total of 1618 LHs were performed, 16 % total laparoscopic hysterectomies and 84 % laparoscopic supracervical hysterectomies. Overall outcomes included mean (SD±) blood loss 108.9 ± 69.2 mL, mean operative time 95.4 ± 39.7 min and a complication occurred in 76 (4.7 %) of cases. Suboptimal perioperative outcomes of an individual surgeon were not always detected on a hospital level. However, collective suboptimal outcomes were faster detected on a hospital level compared to individual surgeon’s level. Evidence of a learning curve is seen; for the first 100 procedures, a decrease in operative time is observed as individual surgeon experience increases. Similarly, the risk of conversion decreases up to the first 50 procedures.

**Conclusion:**

An individual outlier (i.e., surgeon with consistently suboptimal performance) will not always be detected when monitoring outcome measures only on a hospital level. However, monitoring outcome measures on a hospital level will detect suboptimal performance earlier compared to monitoring only on an individual surgeon’s level. To detect performance outliers timely, insight into an individual surgeon’s outcome and skills is recommended. Furthermore, an experienced surgeon is no guarantee for acceptable surgical outcomes.

## Introduction

In an effort to improve patient safety in gynecologic surgery, there has been an increasing focus on measures of perioperative outcomes. As the field of minimally invasive surgery involves new and evolving technology, these procedures may be particularly vulnerable to adverse incidents [1]. Individual surgeon outcomes as well as hospital-wide complication rates have been reported; possible uses for this information vary from quality improvement projects, credentialing, ranking list and reimbursement profiles [2]. One of the main problems of this widely released data is the lack of an accurate case-mix correction (patient characteristics that could influence outcomes). As referral hospitals perform more complex procedures and treat more challenging patients, this can potentially result in less optimal surgical outcomes [3]. This case-mix correction may be appropriate when analyzing data on a surgeon level as well, and has been recommended for parameters including uterine weight and BMI regarding laparoscopic hysterectomy (LH) [3]. In addition, many of the quality assessment registries focus only solely on hospital outcome measures, merging all individual surgeon outcomes. This can result in the lack of detection of lesser-skilled surgeons who may exhibit suboptimal performance. Furthermore, the experience of a surgeon is increasingly being used as a component in the assessment of surgical quality [4–8], and it is important to determine the value of an individual surgical skills factor [9].

The aim of this study is to compare hospital outcome measures versus individual surgeon outcomes for LH. Further, we aim to assess the relationship between surgeon experience and perioperative outcomes once corrected for case-mix characteristics.

## Materials and methods

In this retrospective study, all consecutive cases of laparoscopic hysterectomy (laparoscopic supracervical hysterectomy (LSH) and total laparoscopic hysterectomy (TLH) performed for benign uterine disease between January 2003 to December 2010 at the Department of Obstetrics and Gynecology of the University of Tübingen, Germany were collected. Exclusion criteria included indication of malignancy, deep infiltrating endometriosis or urogenital prolapse in order to limit confounding factors which may be attributed to more complex operations.

The primary outcome measures included: operative time (minutes from first incision to skin closure), estimated blood loss (milliliters) and complications. The blood loss was calculated using the following formula: ((Hemoglobin concentration preoperative (g/l)) − (Hemoglobin 1st day postoperative (g/l)))/((Hemoglobin preoperative (g/l)) − (Hemoglobin 1st day postoperative (g/l)))/2) × 1000 [10]. Complications included infection (local, organ and/or systemic), injury (vascular, bowel, bladder and/or ureter), wound dehiscence, hemorrhage (defined as >1000 mL or post-operative bleeding), thromboembolism formation, organ dysfunction (e.g., urinary retention or incontinence, ileus, liver or kidney dysfunction), systemic events (e.g., medication error, adverse drug reaction, etc.), technical complications (e.g., failed procedure, corpus alienum, etc.), and other (i.e., not specified) [11]. For this study, complications were classified by two levels of severity: level 1 (recovery without (re)operation) and level 2 (reoperation indicated, permanent injury and/or function loss or death). Additional data, which were abstracted from the medical record, included: conversion to laparotomy, BMI (kg/m^2^), uterus weight (gram), number of previous abdominal surgery and age.

The Ethics Committee of the Medical Faculty of the University of Tübingen approved this study.

### Data analysis

Statistical analyses were performed using R statistical software, version 20 for Windows and SPSS version 22 (IBM Corp., Armonk, NY). In addition to descriptive statistics, we fitted regression models for the primary outcomes measures. For the numerical outcomes of blood loss and operative time, a gamma regression model with the logarithmic link function was used. For the categorical outcome of perioperative complications (defined as none, level 1 or level 2) a multinomial regression model with cumulative logistic link function was used. Adjustment factors were adapted from previous research [9]; all outcomes were adjusted for uterine weight. In addition, blood loss was adjusted for BMI and complication was adjusted for the number of previous abdominal surgeries. We computed a numerical complication score by rating a level 1 complication at 1 point and a level 2 at 2 points.

Upon fitting the regression models, we obtained expected outcomes (given the relevant patient characteristics) for each surgery. From these, we constructed individual performance graphs [cumulative Observed minus Expected (O − E)] for every surgeon per surgical outcome (operative time, blood loss and complication score). These individual O − E graphs provided an intuitive representation of the performance in risk-adjusted outcomes over time. Furthermore, we combined the results of all surgeons into a single O − E graph to show the performance at the hospital level. It should be noted, that since we determined the expected performance on the same data, the perceived performance will be exactly according to the benchmark. However, the combined graph shows the progression over time.

Furthermore, we studied the learning effect by regressing the three outcomes on each surgeon’s experience (i.e., number of previous LH performed) in addition to the above-mentioned patient characteristics. We modelled the effect of experience using penalized regression splines as implemented in the R package mgcv [12].

## Results

A total of 1618 LHs were performed by 12 gynecologists over the study period. Overall mean (±SD, range) blood loss was 108.9 (±69, 709)mL, mean operative time 95.4 (±39.7, 390) minutes and there was a 4.7 % complication rate. The surgical experience of the 12 gynecologists ranged between 18 and 202 procedures at the end of the study period. Table 1 outlines the perioperative characteristics of the LH cases by individual surgeon.

**Table 1:** Surgical data of total performed laparoscopic hysterectomies and procedure data per individual surgeon

	Total LHs (*n* = 1618)	Surgeon #1 (*n* = 113)	Surgeon #2 (*n* = 181)	Surgeon #3 (*n* = 202)	Surgeon #4 (*n* = 187)	Surgeon #5 (*n* = 195)	Surgeon #6 (*n* = 184)
BMI, kg/m^2^ (SD)	25.4 (5.0)	25.9 (4.9)	25.7 (5.3)	25.1 (4.8)	25.9 (5.4)	25.6 (5.4)	25.2 (4.8)
Age, years (SD)	53 (6.9)	52 (5.5)	54.4 (6.8)	54.1 (6.5)	52.1 (5.8)	52.5 (6.2)	52.6 (6.1)
Uterus weight, gram (SD)	217.6 (91.0)	212 (178)	200.8 (155.8)	187.5 (134.6)	226.5 (180.6)	233.4 (212.5)	232.7 (194.2)
Previous surgery %							
None	35.6	34.9	32.4	34.9	38.1	35.5	37.2
One	31.3	34.8	35.8	38.5	25.4	29.6	22.1
Two	19.0	21.1	19.9	13.3	16.6	23.8	21.5
>Two	14.1	9.2	11.9	13.3	19.9	11.1	19.2
Blood loss, mL (SD, range*)*	108.9 (69.2, 709)	106.6 (67.3, *309*)	105.9 (61.9, 338)	103.7 (60.0, 352)	113.8 (70.1, 462)	99.0 (68.5, 457)	111.7 (93.5, 709)
Operative time, min (SD, range)	95.4 (39.7, 390)	74.7 (31.8, *181)*	93.7 (35.9, 210)	94.8 (30.0, 170)	93.7 (39.6, 240)	86.5 (38.7, 290)	99.2 (39.5, 221)
Complications %	4.7	1.8	2.8	3.0	4.8	3.1	4.4
Conversion rate %	2.9	1.8	1.7	2.5	1.6	3.1	2.2
Type hysterectomy %							
LSH	84	77.9	87.3	91.1	75.9	79.5	84.2
TLH	16	22.1	12.7	8.9	24.1	20.5	15.8

	Surgeon #7 (*n* = 197)	Surgeon #8 (*n* = 146)	Surgeon #9 (*n* = 132)	Surgeon #10 (*n* = 18)	Surgeon #11 (*n* = 42)	Surgeon #12 (*n* = 21)
BMI, kg/m^2^ (SD)	24.6 (4.2)	26.1 (5.3)	25.2 (4.6)	25.6 (4.1)	25.8 (6.7)	23.3 (3.2)
Age, years (SD)	54.3 (7.6)	52.2 (7.6)	54.7 (7.2)	56.3 (8.6)	55.6 (6.2)	60.3 (10.6)
Uterus weight, gram (SD)	221.3 (225.7)	217.2 (173.0)	246.5 (235.8)	179.8 (112.1)	203.4 (142.8)	177 (149.9)
Previous surgery %						
None	39.4	36.2	37.2	37.5	31.7	28.6
One	32.1	29.8	28.7	18.8	34.1	38.1
Two	17.1	17.0	18.6	31.3	24.4	23.8
>Two	11.4	17.0	15.5	12.4	9.8	9.5
Blood loss, mL (SD, range)	111.7 (63.9, 342)	114.9 (59.6, 378)	110.7 (62.3, 313)	98.4 (76.6, 270)	97.0 (80.8, 342)	142.8 (99.4, 454)
Operative time, min (SD, range)	102.9 (44.3, 228)	111.1 (51.3, 350)	95.6 (36.2, 285)	123.6 (58.3, 243)	89.8 (42.7, 211)	105.0 (37.6, 161)
Complications %	6.1	2.8	4.6	0.0	7.1	0.0
Conversion rate %	5.1	1.4	3.8	0.0	2.4	28.6
Type hysterectomy %						
LSH	91.4	71.9	84.1	100	83.3	90.5
TLH	8.6	28.1	15.9	0	16.7	9.5

**Fig. 1:**
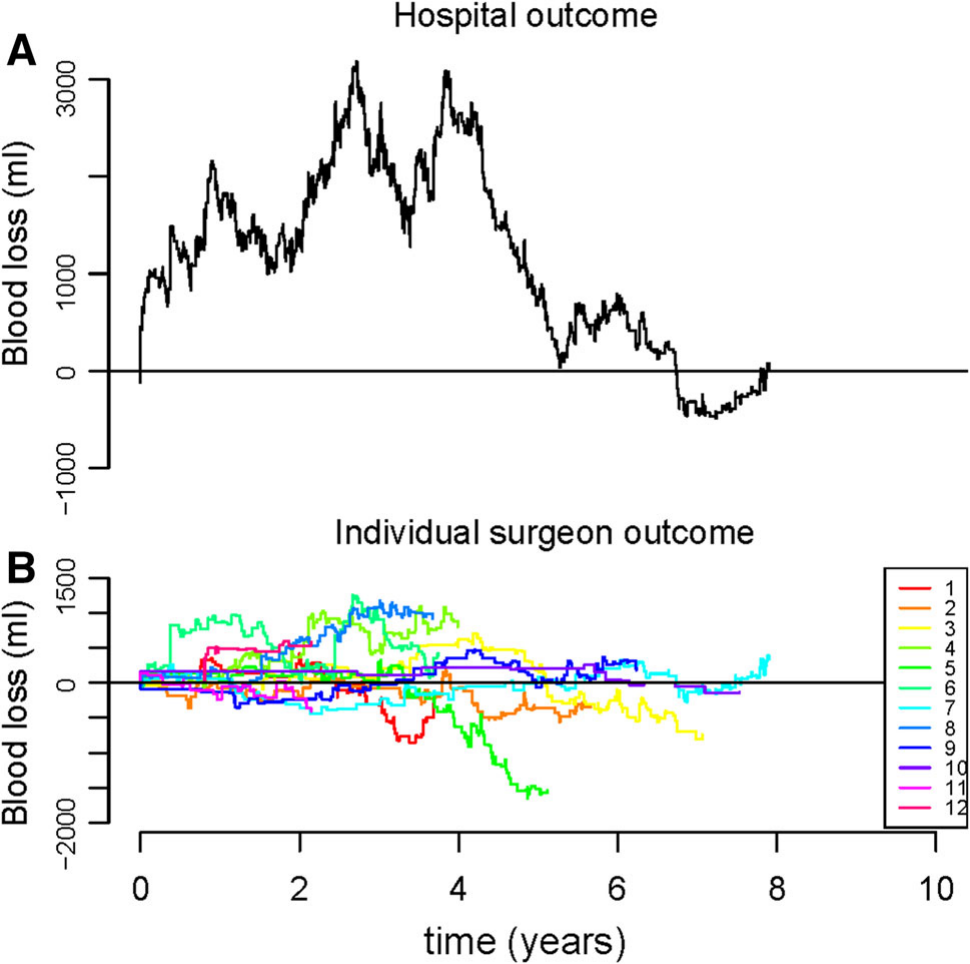
**a** and **b** Observed minus Expected (O − E) graphs for outcome blood loss. Explanation of the graphs: when the *line* drops, the surgeon/hospital performed better than expected. When the *line* rises, the surgeon/hospital performed less optimal than expected

**Fig. 2:**
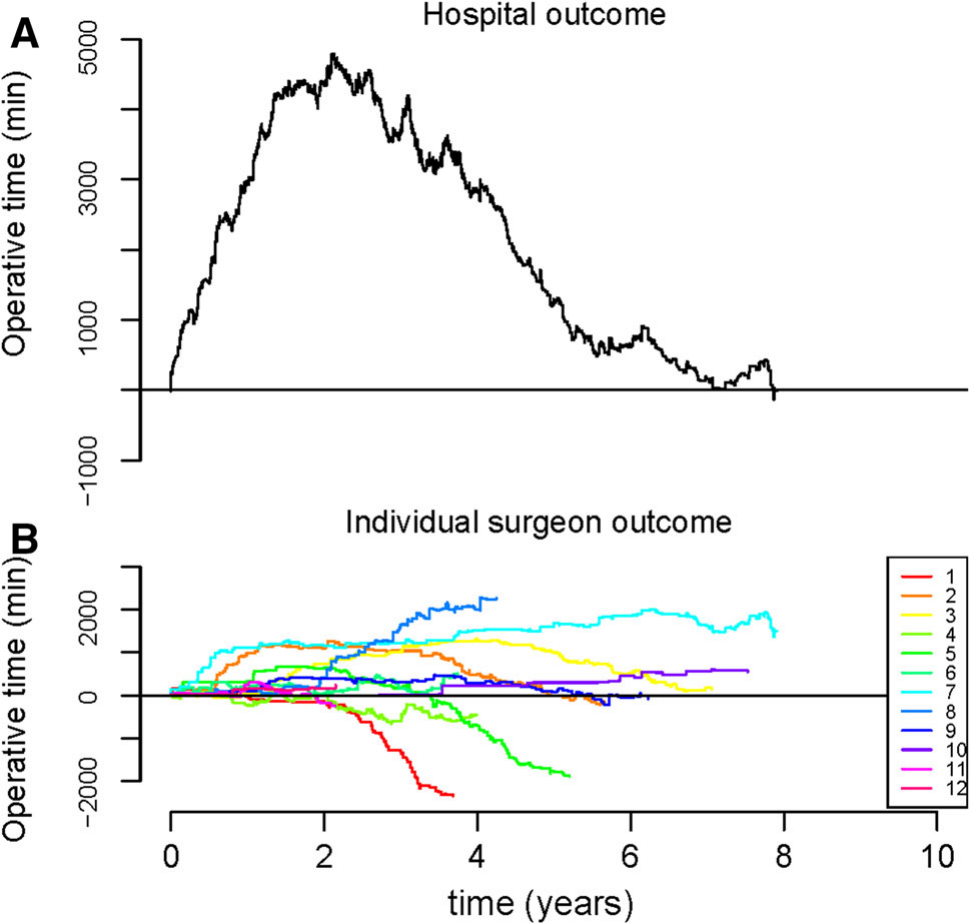
**a** and **b** Observed minus Expected (O − E) graphs for outcome operative time. Explanation of the graphs: when the *line* drops, the surgeon/hospital performed better than expected. When the *line* rises, the surgeon/hospital performed less optimal than expected

**Fig. 3:**
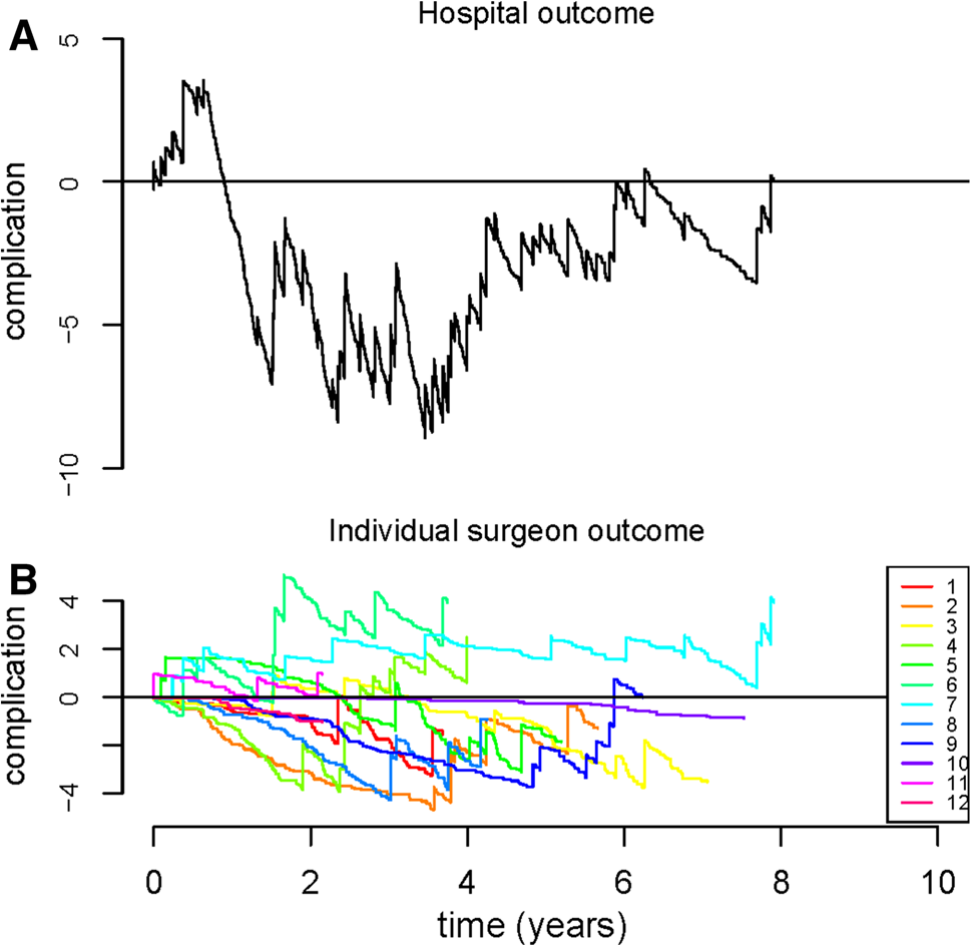
**a** and **b** Observed minus Expected (O − E) graphs for outcome complication score. Explanation of the graphs: when the *line* drops, the surgeon/hospital performed better than expected. When the *line* rises, the surgeon/hospital performed less optimal than expected

Figures 1, 2, 3 show the cumulative Observed minus Expected Graphs for the individual surgical outcome of blood loss, operative time and complication score on both the hospital level (Figs. 1a, 2a, 3a) and the individual surgeon’s level (Figs. 1b, 2b, 3b).

### Hospital-level outcome measures (Figs. 1a, 2a and 3a)

For blood loss (Fig. 1a), the outcome measures were diverse and the graph line alternately moved downward and upward. The downward part of the graph line indicated a cumulative better outcome than expected; the upward part of the graph line indicated a cumulative less optimal outcome than expected.

For operative time (Fig. 2a), less optimal outcomes were observed for the first 2 years, indicating a learning curve. After 2 years a cumulative operative time of 4900 min more than expected was observed. Thereafter, the graph line continued to move downward, indicating that cumulative better outcomes for this hospital were observed than expected.

For complications (i.e., level 1 and level 2 complications) (Fig. 3a), in the first year there was an upward trend in the graph, which indicated less optimal outcomes, with cumulative 3.9 complications more than expected. Thereafter, the graph line moved downward and the complication outcome measure for the hospital continued below zero, indicating that the complication score for the hospital was better than expected.

Comparing individual versus hospital outcome measures, a more rapid detection of suboptimal outcomes was detected for all three outcomes on hospital level (Figs. 1, 2, 3).

### Individual outcome measures (Figs. 1b, 2b, 3b)

For blood loss (Fig. 1b), a considerable difference between all individual outcome measures was observed. Surgeon 8 can be considered an outlier, since the graph of this surgeon continued to move upward (ended with cumulative 915 mL more blood loss than expected). The same applied for surgeon 4 (ended with cumulative 873 mL more blood loss than expected). The best individual outcome measure for blood loss was observed for surgeon 5 (cumulative 1537 mL blood loss less than expected).

With regards to operative time (Fig. 2b), an upward trend in the graphs of almost all individual surgeons was observed for the first 2 years, indicated less optimal performance. Thereafter, most of the surgeons performed better than expected, indicated by a descending graph line. However, surgeon 8 was observed as an outlier, as the graph of this surgeon continued to move upward (ended with cumulative 2267 min more operative time than expected). Surgeon 1 and surgeon 5 can be considered as better skilled surgeon of this hospital, and these outcomes compensated the suboptimal outcome of surgeon 8 (resulting in good outcome measures on a hospital level; i.e., descending graph, Fig. 2a).

For complication score (Fig. 3b), three inferior outliers were observed (surgeon 4, surgeon 6 and surgeon 7) with a score of, respectively, 2.5, 3.9 and 3.92 more complications than expected. The graph line of these surgeons continued to move upward.

### Surgeon’s experience

**Fig. 4:**
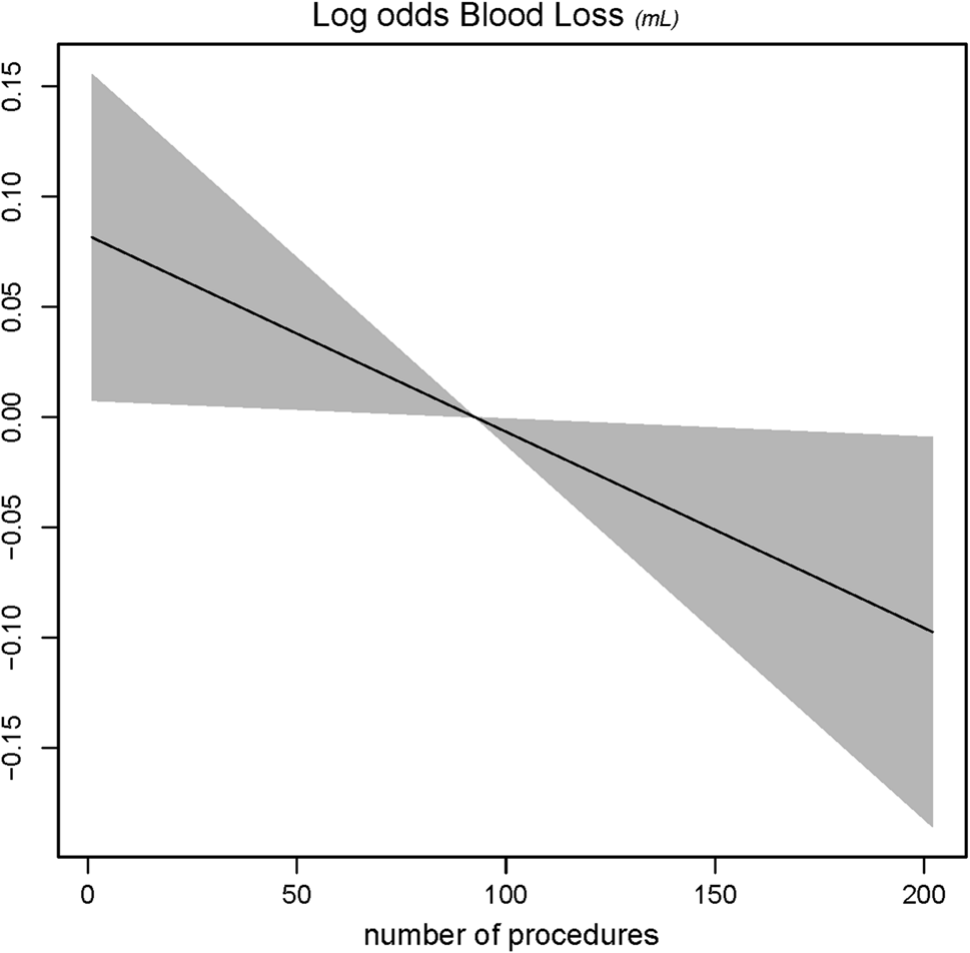
Log odds of blood loss and surgeon’s experience. *The gray shaded area* represents the standard deviation (SD)

**Fig. 5:**
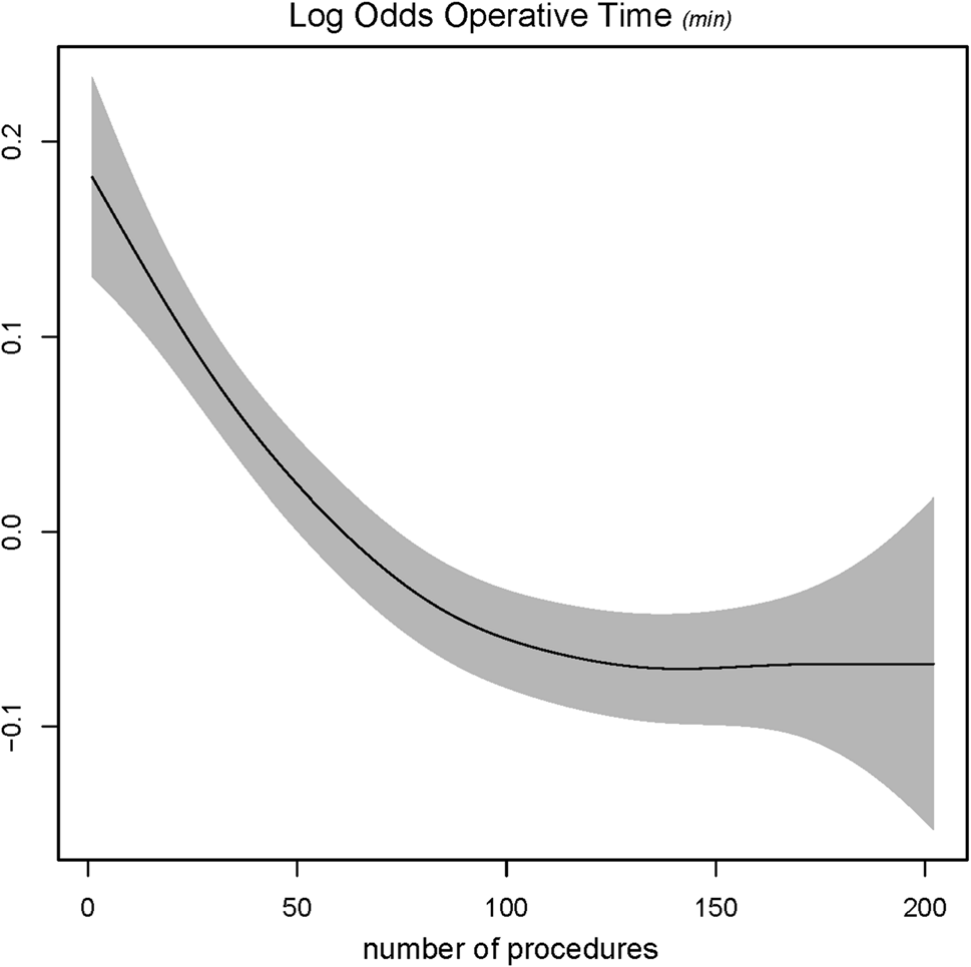
Log odds of operative time and surgeon’s experience. *The gray shaded area* represents the standard deviation (SD)

**Fig. 6:**
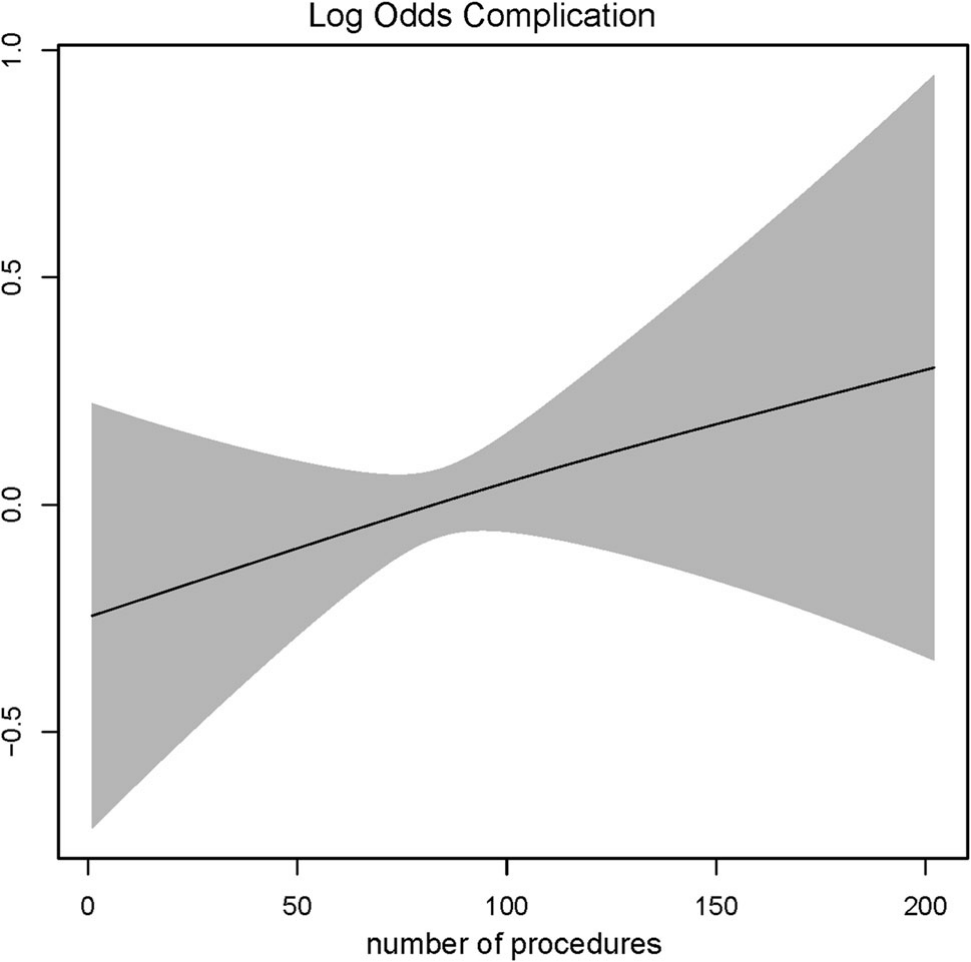
Log odds of complication score and surgeons experience. *The gray shaded area* represents the standard deviation (SD)

**Fig. 7:**
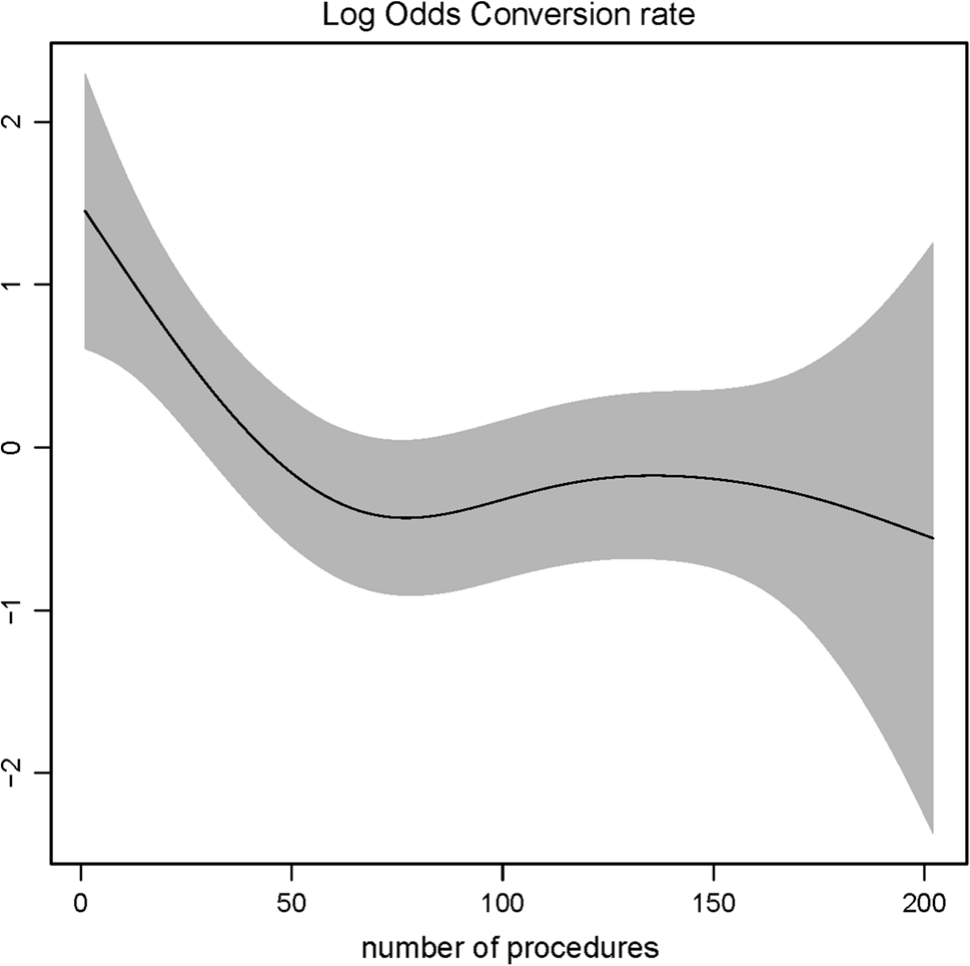
Log odds of conversion rate and surgeons experience. *The gray shaded area* represents the standard deviation (SD)

Figures 4, 5, 6, and 7 showed the log odds graphs of surgeon’s experience per surgical outcome, corrected for case-mix characteristics. For blood loss, an association was observed between increasing surgical experience and decreased blood loss; however, this should be interpreted with caution given the large standard deviation observed (Fig. 4).

For operative time, up to 100 procedures a clear decrease was observed as experience increased (Fig. 5). A higher complication rate was found when experience increased; however, this was not statistically significant (Fig. 6). Up to 50 procedures a clear decrease was observed for conversion rate, with a plateau thereafter (Fig. 7).

## Discussion

Surgeons and hospitals may be expected to provide evidence of the quality of care which they deliver by documenting outcome measures [13]. To date, most of the publically reported quality indicators are based on hospital-level outcome measures, such as complication and reoperation rates. As demonstrated in our results, monitoring outcome measures exclusively on the hospital level will not always detect individual surgeon with extreme outcomes. We have demonstrated that suboptimal outcomes of a lesser-skilled surgeon will be compensated by the superior skills of other surgeons in the same hospital, resulting in a normal or good quality outcome measure for the hospital (Figs. 2, 3, e.g., surgeon 8 is compensated by surgeon 1 and surgeon 5). Therefore, to evaluate quality of care accurately, outcome measures should also be assessed on individual surgeon’s level.

As we observed, good hospital outcome measures do not necessarily reflect good surgeon outcome measures and vice versa. However, when all surgeons of one hospital perform less optimal, this will be detected quicker on a hospital level (Fig. 2). This can be considered as strength of monitoring outcome measures on a hospital level instead of individual.

Surgical experience is often discussed as a proxy for quality assessment measurement [4–8]. Our data also showed a clear association between increased surgical experience and both a decreased operative time (after 100 procedures) and conversion rate (after 50 procedures). Compared to previous literature which has suggested a learning curve of 30 cases for LH, this demonstrates a slower rate of improvement [5, 14]. One possible explanation for the longer learning curve found in this study is that a more experienced surgeon may take on more complex procedures, which can consequently cause more complications and less optimal outcomes [4]. The outcomes in this study were corrected for case-mix characteristics such as uterine weight, BMI and previous abdominal surgery, although there may be unknown variables for which no correction was applied such as severe endometriosis, age and other comorbidities [3]. Hence, our data suggest that experience alone is not sufficient to assure the quality of surgical care; individual skills may provide more information about the actual quality of individual surgical performance.

Strengths of this study include the correction for case-mix characteristics in all performed analyses, which makes the comparison of surgical outcomes more precise. Additionally, we were able to longitudinally follow all 12 surgeons and record all their consecutive procedures from the beginning of their (laparoscopic) career. A potential limitation of our study was the necessity to calculate blood loss using the value of Hemoglobin drop, as opposed to surgeons estimated blood loss or a different objective marker. Furthermore, it is difficult to confirm external validity of the complication rates as our chosen definition of complications differs from the more frequently reported Clavien Dindo scale. Other limitations inherent to the study of quality and performance include the issues of rare outcomes and small case numbers. For example, if the incidence of a particular adverse outcome is relatively low, one can not presume that the absence of a complication in a small series of patients implies optimal care [15]. This phenomenon occurred in our results; two surgeons had a complication rate of 0 % (surgeon 10 and 12), which was based on only a few procedures (18 and 21 procedures, respectively). Additionally, if we look closer to the surgeon with the highest mean operative time (surgeon 10), this was based on 18 procedures and the high mean was only due to one single procedure with an operative time of 284 min. Therefore, small sample sizes should always be taken into account when measuring surgical quality [15]. Small sample size is in general a problem in (advanced) gynecologic surgery [16].Therefore, surgical outcomes with a low incidence should be measured on both hospital level and individual level in an effort to detect consistently suboptimal performance timely.

An important subject for future research is the definition of a performance outlier. Different methods are defined to determine an outlier [17]. In our study we choose to define the outliers as the best and worst performers, compared to their own benchmark. However, this does not necessarily mean that these surgeons are also superior or inferior skilled compared to the national or worldwide benchmark. Therefore, before drawing any conclusion of quality assessment outcomes, benchmark and outlier definition should be defined first, and we urge that international definitions should be adopted. In addition, it is also important to define clinically relevant quality outcomes since, for example, blood loss of 50–100 mL more or less is not always clinically relevant for the patient, and the same applies for operative time. However, recent studies have shown significant associations between increased operative time and complication rates or reoperations [18].

Although performance ratings may be useful, there is potential for falsely low or high ratings both on the surgeon and hospital level. For this reason, reliable case-mix adjustment is of major importance to benchmark surgical outcomes correctly. Our study showed that measurement of quality on a hospital level would detect suboptimal performances quicker and in a more consistent fashion. However, it is still possible to misidentify an individual surgeon who is either a high or low performer. Further insight into the individual surgeon’s outcome measures and skills is required to detect suboptimal performances timely. Furthermore, experience alone is not a sufficient measurement assessment to assure surgical quality and a very experienced surgeon is unfortunately no guarantee for acceptable surgical outcomes.
